# The study of calcified atherosclerotic arteries: an alternative to evaluate the composition of a problematic tissue reveals new insight including metakaryotic cells

**DOI:** 10.1186/s12907-016-0036-6

**Published:** 2016-07-29

**Authors:** Silvia Fittipaldi, Francesco Vasuri, Alessio Degiovanni, Rodolfo Pini, Mauro Gargiulo, Andrea Stella, Gianandrea Pasquinelli, William G. Thilly, Elena V. Gostjeva

**Affiliations:** 1 Department of Experimental, Diagnostic and Specialty Medicine (DIMES); S. Orsola-Malpighi Hospital, Bologna University, Via Massarenti 9, I 40139 Bologna, Italy; 2 Laboratory in Metakaryotic Biology (LIMB), Department of Biological Engineering, Massachusetts Institute of Technology, Cambridge, MA USA

**Keywords:** DNA quantification, Atherosclerosis, Calcification, Osteogenesis, Immunohistochemistry, Metakaryotic cells

## Abstract

**Background:**

Calcifications of atherosclerotic plaques represent a controversial issue as they either lead to the stabilization or rupture of the lesion. However, the cellular key players involved in the progression of the calcified plaques have not yet been described. The primary reason for this lacuna is that decalcification procedures impair protein and nucleic acids contained in the calcified tissue. The aim of our study was to preserve the cellular content of heavily calcified plaques with a new rapid fixation in order to simplify the study of calcifications.

**Methods:**

Here we applied a fixation method for fresh calcified tissue using the Carnoy’s solution followed by an enzymatic tissue digestion with type II collagenase. Immunohistochemistry was performed to verify the preservation of nuclear and cytoplasmic antigens. DNA content and RNA preservation was evaluated respectively with Feulgen staining and RT-PCR. A checklist of steps for successful image analysis was provided. To present the basic features of the F-DNA analysis we used descriptive statistics, skewness and kurtosis. Differences in DNA content were analysed with Kruskal-Wallis and Dunn’s post tests. The value of *P* < 0.05 was considered significant.

**Results:**

Twenty-four vascular adult tissues, sorted as calcified (14) or uncalcified (10), were processed and 17 fetal tissues were used as controls (9 soft and 8 hard). Cells composing the calcified carotid plaques were positive to Desmin, Vimentin, Osteocalcin or Ki-67; the cellular population included smooth muscle cells, osteoblasts and osteoclasts-like cells and metakaryotic cells. The DNA content of each cell type found in the calcified carotid artery was successfully quantified in 7 selected samples. Notably the protocol revealed that DNA content in osteoblasts in fetal control tissues exhibits about half (3.0 ng) of the normal nuclear DNA content (6.0 ng).

**Conclusion:**

Together with standard histology, this technique could give additional information on the cellular content of calcified plaques and help clarify the calcification process during atherosclerosis.

**Electronic supplementary material:**

The online version of this article (doi:10.1186/s12907-016-0036-6) contains supplementary material, which is available to authorized users.

## Background

Considered for decades a passive process, calcification is now seen as an active mechanism sharing many similarities with embryonic bone formation [1, 2]. The most recent mechanism proposed to elucidate arterial calcification is the possible role of resident or circulating stem cells that differentiate into chondro-osteogenic cells [3]. Nowadays, the clinical impact of arterial calcification is still unclear [4]; indeed the extent of calcification is associated with either a good or bad prognosis. For example, in the coronary arteries small calcifications increase the risk of plaque rupture whereas bigger deposits seem to stabilize the plaque [5]. On the other hand, calcified carotid plaques are considered a low athero-embolic risk [6]. In our recent study, we observed that plaques with massive calcifications showed the same incidence of histological complications but without influencing clinical symptomatology [7].

A big issue in analysing calcified plaques is the deterioration of the carotid tissues due to the strong pre-treatment used to dissolve minerals (ethylenediaminetetraacetic acid (EDTA) or chloride acid (HCl) prior to histopathological processing [8].

Previous studies attempted to process calcified tissue with alternative decalcification solutions or treatments as ultrasound, however the major issue was maintaining the whole cellular morphology together with an intact DNA content [9, 10]; indeed the use of strong acids hydrolyses the DNA molecule. We hereby apply a parallel approach to standard histology methods to preserve and study the cellular content of heavily calcified plaques. Our aim was to validate and optimized the techniques for the study of calcified arterial tissues. Additionally to calcified plaques, the technique was also tested on a wide series of tissues from soft to hard (bone).

## Methods

### Tissue sampling

All samples were obtained under a protocol approved in advance by the Massachusetts Institute of Technology, Committee on the Use of Humans as Experimental Subjects. No persons, including minors/children, were enrolled in MIT studies. Only surgical discards from patients anonymous to MIT researchers were received from collaborating hospitals. These comprised fixed tissues from a variety of organs. The Massachusetts Institute of Technology IRB reviewed and approved the several collaborative relationships specifically indicating that the arrangements were in the US NIH “exempt” category for the requirement for informed consent (COUHES approval number 0804002679). Anonymous surgical discards were fixed immediately upon surgical removal in Carnoy’s solution as described and stored under refrigeration in 70 % ethanol [11]. A total of 41 tissue samples were finally collected from 26 cases, 16 fetuses and 10 adults.

Experimental parameters were optimized for two different types of samples, soft and hard. Hard adult samples were defined as “*undissectable*” with standard procedures without previous decalcification, commonly in Osteodec, Bio-optical (EDTA, calcium’s chelates, 40 to 60 min dependent on the tissue dimension). Of the 41 tissue samples retrieved, 22 were hard and 19 were soft, sorted in:

- 8 hard fetal samples; 3 femoral, 1 nasal bone, 1 elbow, 1 hip bone and 1 knee bone, and 1 chondroid tissue.
- 9 fetal soft samples; 3 guts, 3 brains, 2 muscles, and 1 eye retinae.
- 14 hard adult samples; 13 calcified carotid plaques and 1 calcified abdominal aortic aneurism.
- 10 soft adult samples; 9 uncalcified carotid plaques and 1 uncalcified abdominal aortic aneurism.

### Spreading method

All 41 tissue samples were fixed in cold Carnoy at 4 °C (3 ml Ethanol 100 % and 1 ml glacial acetic acid per <0.25 cm^2^ within 30 min from surgical removal. Carnoy solution was changed after 60 and 120 min for a total of 3 hours fixation. Sample were then stored at -20 °C in 70 % ethanol [12]. Subsequent enzymatic tissue treatments with collagenase, type II (Clostridium histolyticum, ≥200 collagenase units/mg, Calbiochem, Merck Chemicals Ltd, UK) were tested with different incubation times and concentrations at 37 °C to define satisfactory conditions for various tissue types (Table 1). The most notable requirement is the need for up to 24 h of collagenase treatment to achieve unfolding of the hard tissues.

**Table 1:** Specifications for satisfactory spreading of soft versus calcified tissue samples in adult vascular tissues and fetal samples

Type of tissue	Collagenase II digestion time	Collagenase II working concentration (U^a^/ml)	Acetic acid 45 % maceration tIme	Spread sections size (max)
Adult hard	24 h	10U/1 ml	30 min	0.1 mm
Adult soft	18 h	10U/1.5 ml	10 min	0.3 mm
Fetal hard	12 h	10U/1.5 ml	20 min	0.2 mm
Fetal soft	1–4 h	10U/1.5 ml	10 min	0.3 mm

^a^One CDU unit is defined as the amount of enzyme that will release 1.0 nmol Leu equivalent from collagenase per min at 37 °C, pH 7.2

Collagenase-treated tissue samples were subsequently macerated in 500 ul of 45 % acetic acid (Table 1). Macerated tissue samples were minced with surgical scissors creating pieces no greater than 0.3 mm in length. Each piece was transferred to a slide in a drop of 45 % acetic acid. To spread the macerated tissue into a monolayer, several thicknesses of filter paper were placed on the coverslip and a tweezer handle was moved steadily in one direction with a delicate pressure. Intermittent microscopic examination during the spreading process was used to determine when satisfactory spreading had been achieved. Increased pressure on the stacked filter paper was required for the harder bone-containing samples. Cover slips were removed after direct immersion in liquid nitrogen, and slides were air dried for 1 h. Spread slides were stored at 4 °C, in the dark, and subsequently used for different forms of analyses: Von Kossa, Feulgen-Giemsa staining, immunohistochemistry, Feulgen reaction for DNA quantification and RNA extraction.

### Histochemistry

Histochemistry included Von Kossa staining for the identification of calcified matrix: sections were hydrated, treated with 1.5 % silver nitrate (AgNO3) solution and placed in front of a 100 W lamp for 1 h. Sections were therefore rinsed in distilled H_2_O (dH_2_O), counterstained with nuclear-fast Red, dehydrated and mounted.

The Feulgen-Giemsa staining was used to test the preservation of the nuclei after the collagenase digestion and the subsequent DNA quantification. Briefly, the slides were placed in a Coplin jar filled with HCl 1 N at 60 °C for 8 min, for partial hydrolysis of the macromolecules and DNA depurination. Hydrolysis was stopped by washing the slides in cold dH_2_O. After carefully drying, sections were placed in Schiff’s reagent (#3952016, Sigma-Aldrich, St. Louis MO, US) for 1 h at room temperature, washed twice with 2x standard saline citrate (trisodium citrate 8.8 g/L, sodium chloride 17.5 g/L), washed again with dH_2_O and thereafter counterstained with 1 % Giemsa solution (#GS500, Sigma-Aldrich, St. Louis MO, US) for 5 min. Slides were dehydrated in graded step of alcohol and mounted with Canada Balsam (Sigma- Aldrich C1795).

### Feulgen DNA quantification

Feulgen densitometry relies on the premise that the amount of stain bound to DNA is proportional to the amount of DNA present in the nucleus with a 1:1 ratio. Feulgen reaction is specific for DNA and does not stain RNA [11, 13].

DNA quantification using Feulgen reaction (without Giemsa) was carried out in 3 fetal and 4 adult samples, each equally distributed as hard and soft areas (Table 2, raw data are included in the Additional file 1). Nuclei of chicken red blood cells (cRBC, #IC05-0810, Innovative Research, Novi, MI, US) were used as an internal standard for DNA quantification (one nucleus of RBC contains a diploid quantity of DNA equivalent to 2C = 2.5 pg). Slides are Feulgen stained in groups (25 to 100 slides) with the same reagents, lots, conditions and standards. Image acquisition and segmentation were performed with the Carl Zeiss Vision AxioVision software: these are the critical steps for a precise DNA quantification. All steps are presented in details in the Additional file 2. The measure features for each nucleus are the densitometric sum, the densitometric area and the densitometric standard deviation. The quantity of stain is determined based on the absorbance (Optical Density OD) evaluated by the transmitted light (T).

**Table 2:** Descriptive statistics for Feulgen-DNA content and mean nuclear area

	Origin	Cell type	Nuclei Nr	Skewness	Kurtosis	DNA pg	SD	Mean μm^2^	SD	CV	SE	CI
1	Adult	Hard	137	0.4	-0.6	6.0	3.1	72.7	36.9	50.8 %	3.2	66.5–79.0
2	Adult	Soft	306	1.1	1.8	4.3	2.1	57.5	25.9	45.2 %	1.5	54.6–60.4
3	Adult	Soft	48	1.4	2.8	5.6	2.2	66.1	24.7	36.8 %	3.5	59.0–73.1
4	Adult	Soft	92	0.6	0.5	7.0	2.8	68.4	27.	40.4 %	2.9	62.7–74.1
5	Fetus	Hard	145	1.6	2.7	3.1	1.6	46.9	25.3	53.9 %	2.1	42.7–51.0
6	Fetus	Hard	46	1.5	1.7	1.7	0.9	31.4	16.8	53.5 %	2.5	26.4–36.3
7	Fetus	Soft	120	1.1	0.5	4.8	2.4	63.8	32.7	51.2 %	2.9	57.9–69.7
Total			894					59.2	30.2	51.1 %	1.0	57.2–61.2
cRBC	Control		339	-0.1	1.2	2.5^a^		25.9	5.9	22.6 %	0.3	25.3–26.5

*SD* standard deviation, *CV* coefficient of variation, *SE* standard error, *CI* confidence interval. Characteristics’ of samples: 1, calcified atherosclerotic carotid; 2, soft atherosclerotic carotid; 3, healthy carotid; 4, abdominal aortic aneurism: 5, Femoral bone 11 weeks; 6, chondroid tissue 14 weeks; 7, fetal aorta 15 weeks

^a^Constant known quantity of DNA for the standard cRBC (the chicken red blood cells used as internal standards in each sample). Raw data are presented in the Additional file 1

1 $$ OD\kern0.37em =\kern0.37em lo{g}_{10}\left( 1/T\right) $$

As nuclear DNA stain is heterogeneous, a single point (OD) would not be representative. Thus it is necessary to evaluate the whole nucleus as the integrated optical density (IOD):

2 $$ IOD={\displaystyle {\sum}_{i=1}^nlo{g}_{10}\left( 1/Ti\right)} $$

The scale of each image was 95.04 pixel/ μm^2^. Additionally to nuclear IOD, the measured features were the nuclear area (μm^2^) and the total number of nuclei evaluated.

### Calculation of genome size DNA

To convert IOD in genome size in pg we used the primary standard of RBC (1 C = 1.25 pg). Smears were processed with the same way as samples and were included in each experiment. To calculate DNA content a standard curve (IOD *vs* known C-value) was generated and used to verify that the stain was accurate:

$$ \mathrm{D}\mathrm{N}\mathrm{A}\ \mathrm{pg}\ \mathrm{per}\ \mathrm{nuclei}\ \mathrm{in}\ \mathrm{sample} = 2.5\ \mathrm{pg}\ /\ \mathrm{mean}\ \mathrm{I}\mathrm{O}\mathrm{D}\ \mathrm{standard}\ \mathrm{x}\ \mathrm{I}\mathrm{O}\mathrm{D}\ \mathrm{sample} $$

### Immunohistochemistry technique on Carnoy-fixed spread tissue

Table 3 summarizes the antibody characteristics and the blocking/antigen unmasking procedures used. Indeed, due to the Carnoy fixation, some modifications in the antigen retrieval procedures were optimized.

**Table 3:** Technical characteristics of the antibodies used for imunohistochemistry

Antibodies	Type	Antigen specifications	Company	Locus-Clone	Dilution	Endogenous peroxidase blocking	Antigen unmasking treatment
Ki-67	Mouse Monoclonal	Perichromosomal layer protein. Identifies cells in G1-S-G2-M phases.	Dako A/S, Copenhagen, Denmark	10q26.2 MIB-1	1:100	20 min	Citrate Buffer, heat mediated (4 cycles 750 W, 5 min each)
Osteocalcin	Rabbit Polyclonal	Bone specific protein. Synthesized by osteoblasts, accumulates in the bone matrix.	Millipore USA	1p22	1:100	30 min	Tryton 0.5 % 4 min
Desmin	Mouse Monoclonal	Muscle specific intermediate filament type III protein.	Millipore USA	2q35 D33	1:100	20 min	Citrate Buffer, heat mediated (4 cycles 750 W, 5 min each)
Vimentin	Mouse Monoclonal	Intermediate filament type III protein expressed in mesenchymal cells.	Millipore USA	10p13 V9	1:80	20 min	Not required

In addition to Von Kossa staining, immunostaining for Ostecalcin (OCN) was used to spot the main components of the bone matrix as well as the osteoblasts.

Samples were immunostained for Ki-67 antigen in order to assess the chromatin preservation after the procedure and the cell proliferation. Finally, in order to verify the preservation of specific cytoplasmic antigens, Desmin and Vimentin immunostaining was performed. Carnoy-fixed spread tissue was rehydrated through graded steps of ethanol absolute (100 %, Methanol 3 % H_2_O_2_, 95 %, 70 %, H_2_O). Endogenous peroxidase activity was neutralized with 3 % H_2_O_2_ in absolute methanol at room temperature (rt), in the dark. Antigen-antibody reaction was developed with the NovoLink® Polymer Detection Kit (Novocastra, Newcastle, UK). To reduce nonspecific antibody bindings, hydrophobic binding sites were blocked with Casein 0.4 % (Novolink Protein Block). Sections were incubated 1 h in a wet chamber at rt with the primary antibodies.

To detect primary antibody bound to tissue, sections were incubated with NovoLink® Polymer (8 mg/L) for 30 min at rt. Spreadings were further incubated with chromogen/substrate, 3,3 - diaminobenzidine NovoLink® (DAB pre-diluted 1:20), 30 s for fetal tissue and 2 min for adult tissue, at rt, Cell nuclei were counterstained with Mayer’s haematoxylin (Sigma Chemicals). Fetal tissue was used as positive controls [12]. As negative controls primary antibodies were omitted. Washing steps were all performed with PBS. After dehydration in graded steps of ethanol, samples were mounted onto glass slides using Canada Balsam (Sigma-Aldrich C1795). The sections were observed under a light microscope (Axio Imager Z1 Zeiss, Germany) connected with a charge-coupled device (CCD) camera.

### RNA content: RT-PCR

#### RNA extraction

The RNA was extracted from Carnoy fixed spread sections (monolayer). A commercial kit was used for RNA extraction (RNeasy® FFPE, Cat No. 73504, QIAGEN GmbH, Hilden, Germany); the protocol was slightly modified to increase RNA yield and purity. Spread tissue was recovered from the slides with a scalpel by adding 5 μl of Proteinase K buffer (PKD), under RNase free conditions. Retrieved tissues were combined into a single nuclease-free screw cap tube, dissolved in 245 μl of PKD and followed by centrifugation for 1 min at 10 000 rpm. For cell lysis, we added 20 μL of protease K; the mixture was incubated for 30 min at 56 °C, mixed at 850 rpm, and then incubated again at 80 °C for 15 min. The protocol was followed as stated by manufacturer’s instruction. Finally the RNA was eluted in 22 μl of RNAse free-water, heated at 65 °C for 5 min in order to be denatured and to inactivate RNases, and stored at -80 °C until used. RNA integrity and concentration were measured by using an ND-1000 spectrophotometer (NanoDrop, Fisher Thermo, Wilmington, DE, USA). The ratio of the readings at 260 nm and 280 nm (A260/A280) provides an estimate of the purity of RNA with respect to the contaminants that absorb in the UV spectrum, such as protein. Pure RNA has an A260/A280 ratio of 1.9–2.1.

#### RT-PCR assay

To assess the possibility of extracting RNA from Carnoy-fixed spread tissue as template for retrotranscription and gene expression, we amplified by RT-PCR the housekeeping gene beta-glucuronidase (GUSB) for both femur bone and intestine. GUSB is considered an housekeeping gene required for the maintenance of basic cellular function and expressed in all cells [14].

We also amplified the transcription factor Osterix (OSX) essential for osteoblast differentiation and bone formation [15] and the carcinoembryonic antigen-related cell adhesion molecule 5 (CEACAM 5) normally produced in gastrointestinal tissue during fetal development [16].

The reverse transcription assay was performed using 2 μg of total RNA per 20 μl of mix, following the manufacturer’s protocol (High capacity cDNA Archive kit, Applied Biosystem). The cDNA was stored at -20 °C until RT-PCR was performed. RT-PCR was carried out following MasterMix TaqMan® Protocol (TaqMan Univ PCR MasterMix, Applied Biosystems). Two μl of neat cDNA were amplified using specific probes for GUSB (NM_000181.3), CEACAM5 (NM_004363.2) and Osterix (NM_001173467.1) in the RT-PCR mix (TaqMan® Gene Expression Assay, Applied Biosystems, respective ID assay: Hs00939627_m1, Hs00237075_m1, Hs00541729_m1). Reactions were run on ABI PRISM 7900HT Sequence Detection System (Applied Biosystems). The cycling conditions were performed as follows: 10 min at 95 °C, 45 cycles at 95 °C for 15 s and 60 °C for 60 s. Each assay was carried out in triplicate and the transcription level was normalized using GUSB as a reference gene. The threshold was set at 0.2 in order to be in the exponential phase. The values were expressed as DCT (= > CT Target − CT GUSB).

### Statistics

To present the basic features of the F-DNA analysis we used descriptive statistics: mean, standard deviation, coefficient of variation, standard error, confidence interval stated at 95 %, skewness and kurtosis. Differences in DNA content in pg and fluctuations of DNA (expressed as DNA content variation, DCV) [17] between samples were analysed with Kruskal-Wallis (non parametric-unmatched) and Dunn’s post tests. The value of *P* < 0.05 was considered significant.

## Results

### Immunohistochemistry

Samples of all 41 tissues specifically including the 22 “hard” tissues containing calcified sections were satisfactorily spread for further analyses. Methods of decalcification, i.e. EDTA treatment, was required in tissue processed with the standard procedure but was not required in the spread method using collagenase.

**Fig. 1:**
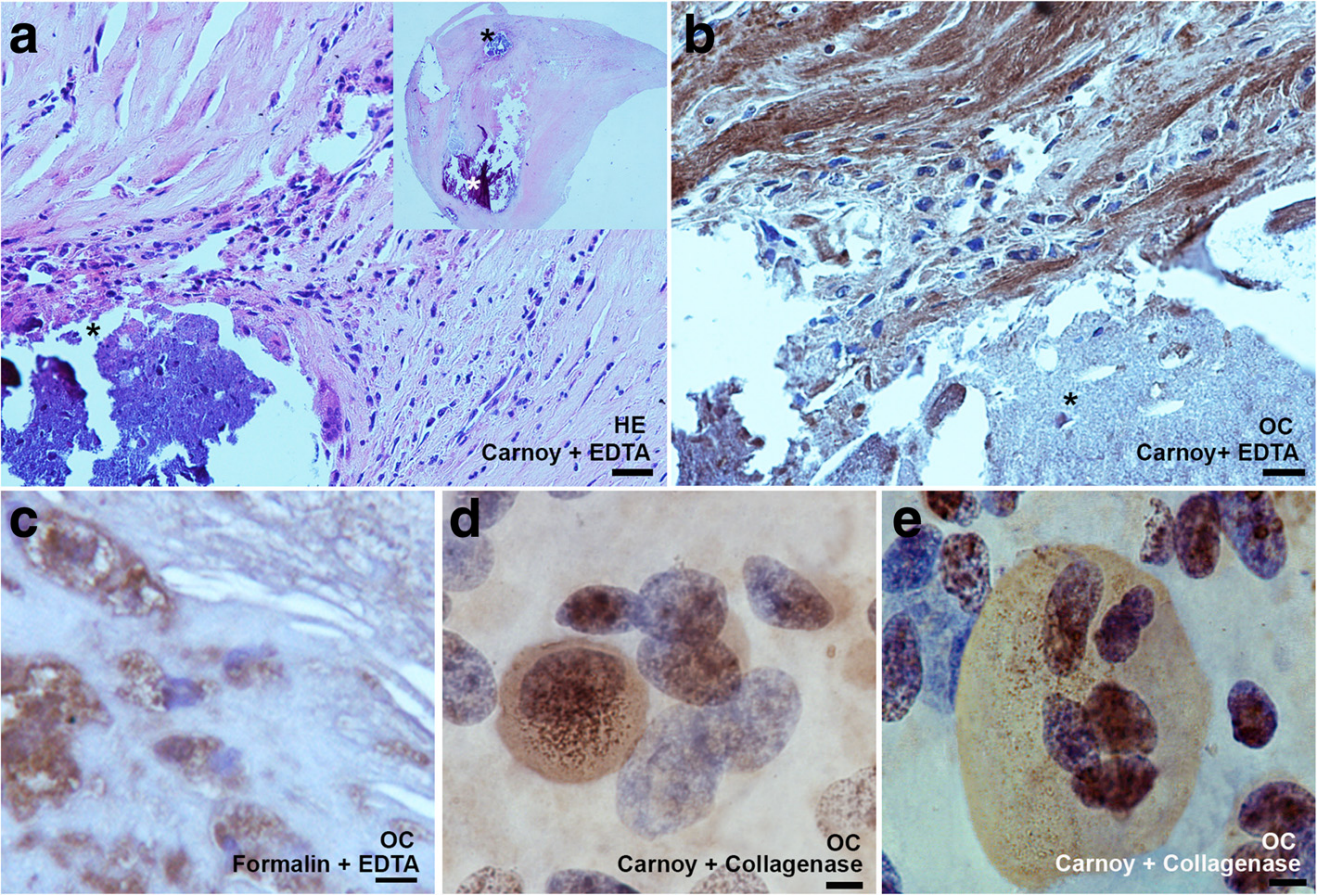
Adult calcified carotid artery sections EDTA treated (**a**-**b**-**c**) and collagenase treated (**c**-**d**). **a** Haematoxylin-Eosin (HE) staining of a standard section fixed in carnoy and EDTA treated (scale bar 40 μm). The insert in figure **a** shows the whole arterial section with a diameter of 4 mm, * asterisks highlights the calcified areas. **b** osteocalcin immunostain of the same tissue fixed in carnoy (scale bar 20 μm) or (**c**) fixed in formalin (scale bar 10 μm). **d**-**e** Osteocalcin immunostaining of the collagenase treated tissue fixed in carnoy. **d** positive osteoblast and (**e**) positive osteoclast-like cells, scale bar 5 μm. Nucleus and cytoplasmatic structure are clearly visible in collagenase spread tissue compared to standard section (**a** and **b**)

The Fig. 1 compared the results obtained with EDTA treatment (Fig. 1a and b) *versus* collagenase digestion of a calcified carotid artery (Fig. 1d and e). Fig. 1a showed a Haematoxylin-Eosin section of calcified artery fixed in carnoy, embedded in paraffin and decalcified with EDTA. Precipitates of calcium were very basophilic so calcification areas were easily visible in violet (asterisk in Fig. 1). In the insert of figure A, we also noticed an empty area (white asterisk), where the calcified material was lost. Figure 1b showed the same tissue of Fig. 1a immunostained for osteocalcin. The Fig. 1c showed the standard section fixed in formalin, EDTA treated and stained for Osteocalcin, while Fig. 1d and e displays the same tissue treated with collagenase and stained for Osteocalcin. The immunohistochemistry (IHC) for Osteocalcin evidenced osteoblasts and synthesized matrix in the adult calcified artery (Fig. 1d and e). All fetal cases and two calcified adult cases showed multinucleated osteoclasts-like cells positive to Osteocalcin. Von Kossa histochemical stain evidenced the same calcified tissues in adult samples, but was negative in fetal samples, where no osteogenic calcification was present yet.

**Fig. 2:**
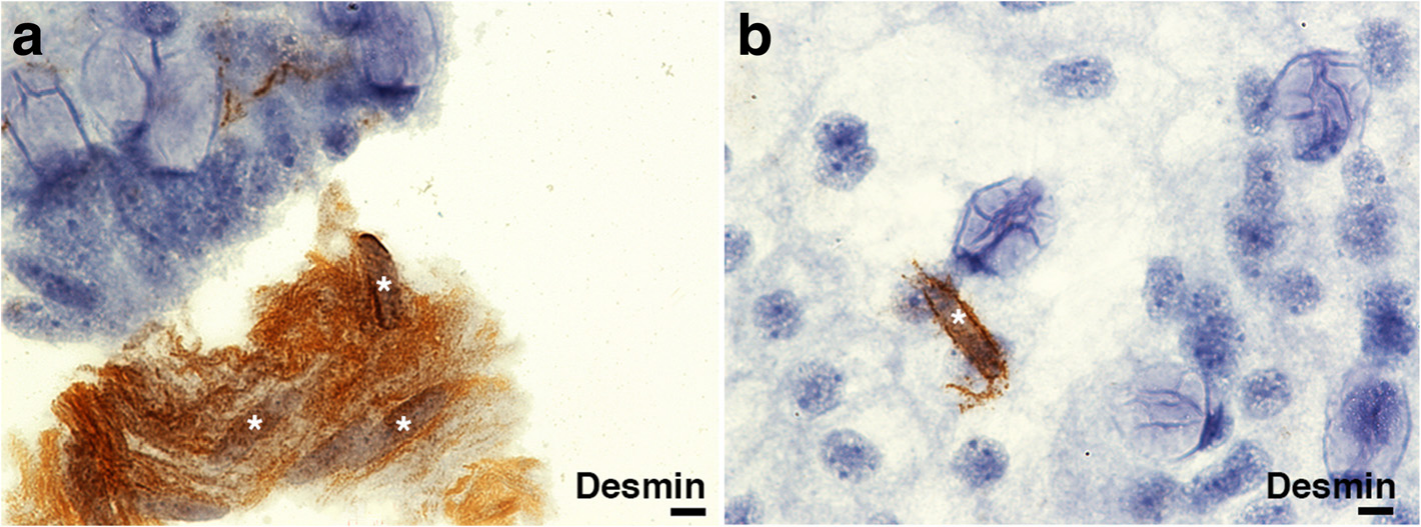
Desmin IHC staining in spread fetal tissue. **a** group of desmin positive smooth muscle cells (SMC) and (**b**) a single SMC in the fetal bowel crypts. *SMC are indicated by a white asterisk and showed the typical elongated “cigar-shaped” nuclei. The SMC along the crypts show a moderate to strong cytoplasmatic staining reaction, scale bar 10 μm

**Fig. 3:**
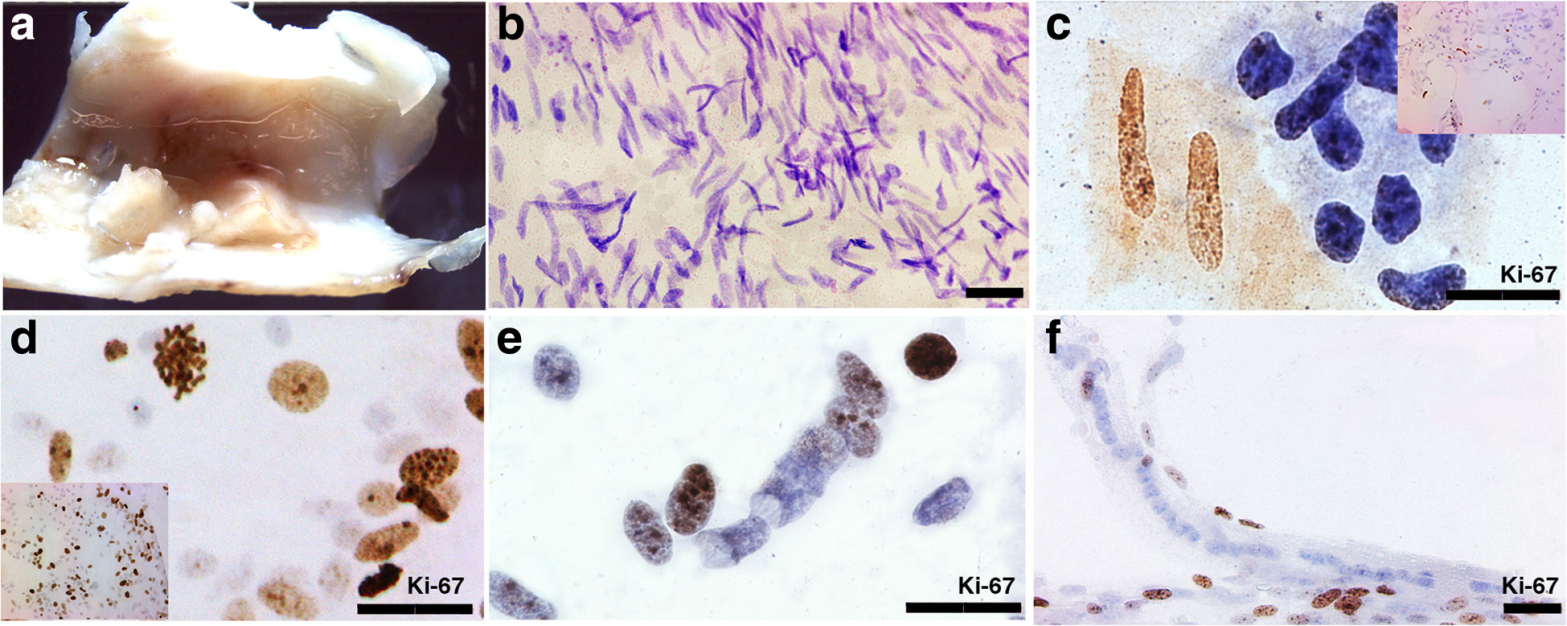
Ki-67 IHC staining in spread tissues. **a** adult calcified artery fixed in Carnoy, length of 1.5 cm. **b** Haematoxylin staining and (**c**-**d**) the corresponding ki-67 staining of the calcified artery at low magnification (inserts) and higher magnification. **e**-**f** positive control for Ki-67 nuclear immunostain (fetal bowel tissue), scale bar 25 μm

Antigen for cytoplasmic intermediates Desmin (Fig. 2) and Vimentin (not shown) were found in all preparations. Desmin and Vimentin showed a reliable positivity indicating that the collagenase digestion of samples and the other treatments did not damage the cytoplasmic intermediate filaments. Figure 2a and B show the good preservation of the smooth muscle cells expressing Desmin in the spread tissue. IHC for Ki-67 confirmed also the good preservation of the chromatin in the spread tissue (Fig. 3c-f). Surprisingly, Ki-67 was also detected in some cells in heavily calcified tissues: for example, case #1 (calcified atherosclerotic carotid, Fig. 3a) was described as acellular during routine IHC analysis by means of standard 2.5 μM sections. However, the monolayer IHC analysis revealed the presence of a heterogeneous population of cells (Fig. 3b), including Ki-67 positive cells (Fig. 3c and d). Mitotic figures were easily spotted with ki-67 staining (Fig. 3d). To verify the specificity of ki-67 staining in the spread artery, a tissue with a high rate of proliferative cells (fetal tissues) was selected and stained with ki-67 (Fig. 3e-f).

### Nuclear DNA content of spread tissues

**Fig. 4:**
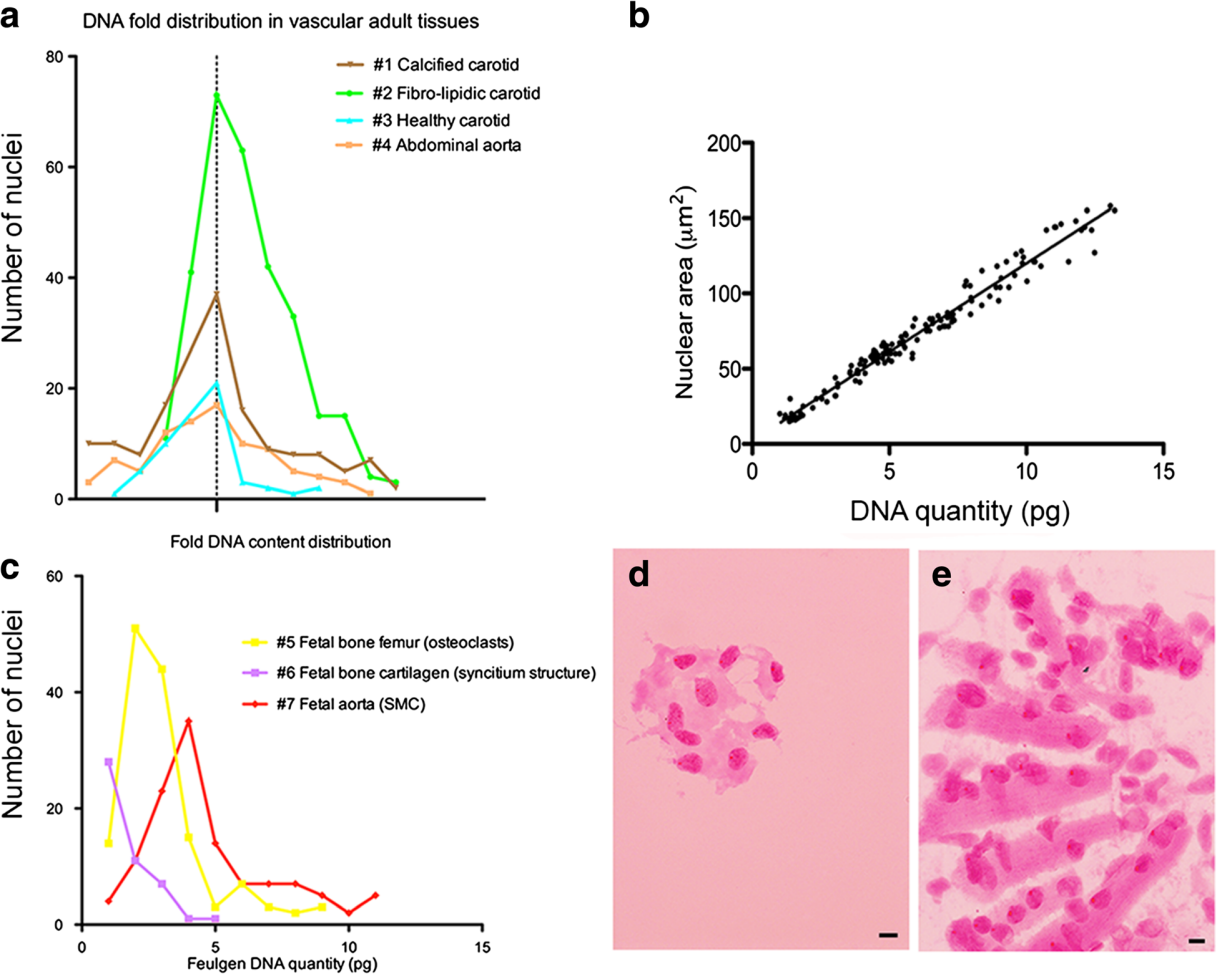
Feulgen DNA analysis (**a**) Unimodal distribution of Feulgen-DNA content expressed as fold DNA content (interrupted lines designate modal positions of diploid 2C), (**b**) Relation between DNA content and nuclear area (**c**) Feulgen DNA quantity (pg) in fetal tissues and (**d**-**e**) examples of the images used for F-DNA quantification. **d** fetal femoral bone femur osteoclasts (#5), (**e**) fetal femoral bone cartilage syncytia (#6), scale bar 5 μm

The Feulgen DNA quantification was performed in the 7 cases listed in Table 2: a total of 894 nuclei were evaluated for IOD and nuclear areas. Overall sample mean IOD was 2.3 ± 1.3 × 10^7, overall mean nuclear area was 59.2 ± 30.2 μm^2^. In the standard cRBC a total of 339 nuclei were examined: mean IOD was 1.2 ± 0.3 × 10^7, mean nuclear area was 25.9 ± 5.9 μm^2^. Feulgen absolute DNA content was successfully calculated in all 7 cases, both soft and hard tissues. In particular, the heavily calcified carotid artery lesion (case #1), had a mean DNA quantity of 6.0 pg, concomitant with the known DNA diploid content of human cells (2C = 6 pg) (Fig. 4a, Table 2). Kurtosis and skewness analysis showed that samples had a unimodal, near-Gaussian distribution of the diploid F-DNA (2C) (Table 2, Fig. 4a). The nuclear size was directly proportional to the DNA content of the cells in the 7 cases. Indeed, Fig. 4b shows that the quantity of DNA within each cell of the calcified carotid artery (#1) was significantly proportional to the area of the nucleus (*R*^*2*^ = 0.97, Pearson correlation *p* < 0.0001). This means that there is a good preservation of the nuclear structures also in the heavily calcified tissues after the procedure.

Adult and fetal tissues showed a significant DNA content variation (DCV, Table 2) within cells, except for DNA content in soft healthy adult arteries (#3, 5.6 pg, CV 36.8 %) compared to DNA content in fetal aortic cells (#7, 4.8 pg, CV 51.2 %). Both samples contained a homogenous population of smooth muscle cells. Of note, soft tissue from healthy carotid arteries #3 had the lowest adult CV (5.6 pg, 36.8 %). DCV significantly differs between pathological atherosclerotic soft arteries (45.2 %) and calcified arteries (50.8 %). Highest DCV were found in all fetal tissues (range 51.2 to 53.9 %).

In particular, two cases showed significant unexpected lower mean DNA content per nucleus compared to adult samples; case #5 (fetal femoral bone) with 3.1 ± 1.6 pg and case #6 (chondroid tissue) with 1.7 ± 0.9 pg (*p* < 0.0001, Table 2, Fig. 4c). In those cases we decided to perform detailed analyses of the DNA in each single cell composing the tissues (the AxioVision software marked each nucleus with a single identification number corresponding to its IOD). We noticed that case #5 was mainly composed of osteoclasts and case #6 of syncytia (Fig. 4d and e). By measuring the DNA content per nucleus we observed unexpected results. Nuclei outside osteoclasts and syncytial formations showed a constant DNA content of 6 pg. In osteoclasts, DNA content per nucleus ranged from 2 to 3 pg. In the syncytia formations, nucleus showed a DNA content ranging from 1 to 2 pg per nucleus. Surprisingly the mean DNA content per syncytium (sum of nuclear DNA nucleus in the whole syncytia) was 6.6 ± 1.4 pg.

### RNA preservation (RT-PCR)

Additionally to the housekeeping gene GUSB, we evaluated the expression of CEA for intestinal fetal tissue and Osterix for calcified/osteogenic tissues. The total mean extracted mRNA from fetal intestine tissue (FI) and femur bone (FB) was 7374 ng and 1430 ng respectively. A260/A280 ratio was 1.94 for both fetal intestine and fetal bone showing the absence of contamination of the RNA extracted (standard ratio range from 1.9 to 2.1). The mean threshold cycle (CT) values of endogenous control GUSB were 30.34 ± 0.18 in FI and 37.34 ± 0.24 in FB. Mean CT for tested gene OSX and CEA were 38.52 ± 0.10 and 35.34 ± 0.31 respectively. ΔCT CEA and ΔCT OSX were 5.02 and 1.18 respectively (Table 4).

**Table 4:** Threshold cycle (Ct) values for GUSB, OSX and CEA in fetal tissues

	Threshold cycle values		
Micro-dissected sample	β-glucuronidase GUSB	Osterix OSX	Carcino-embryonic antigen CEA
Fetal femur bone 12w	37.06	38.42	-
	37.50	38.63	
	37.45	38.51	
Fetal intestine 9w	30.03	-	35.70
	30.32		35.15
	30.37		35.17

### Additional cellular component observed in arteries

**Fig. 5:**
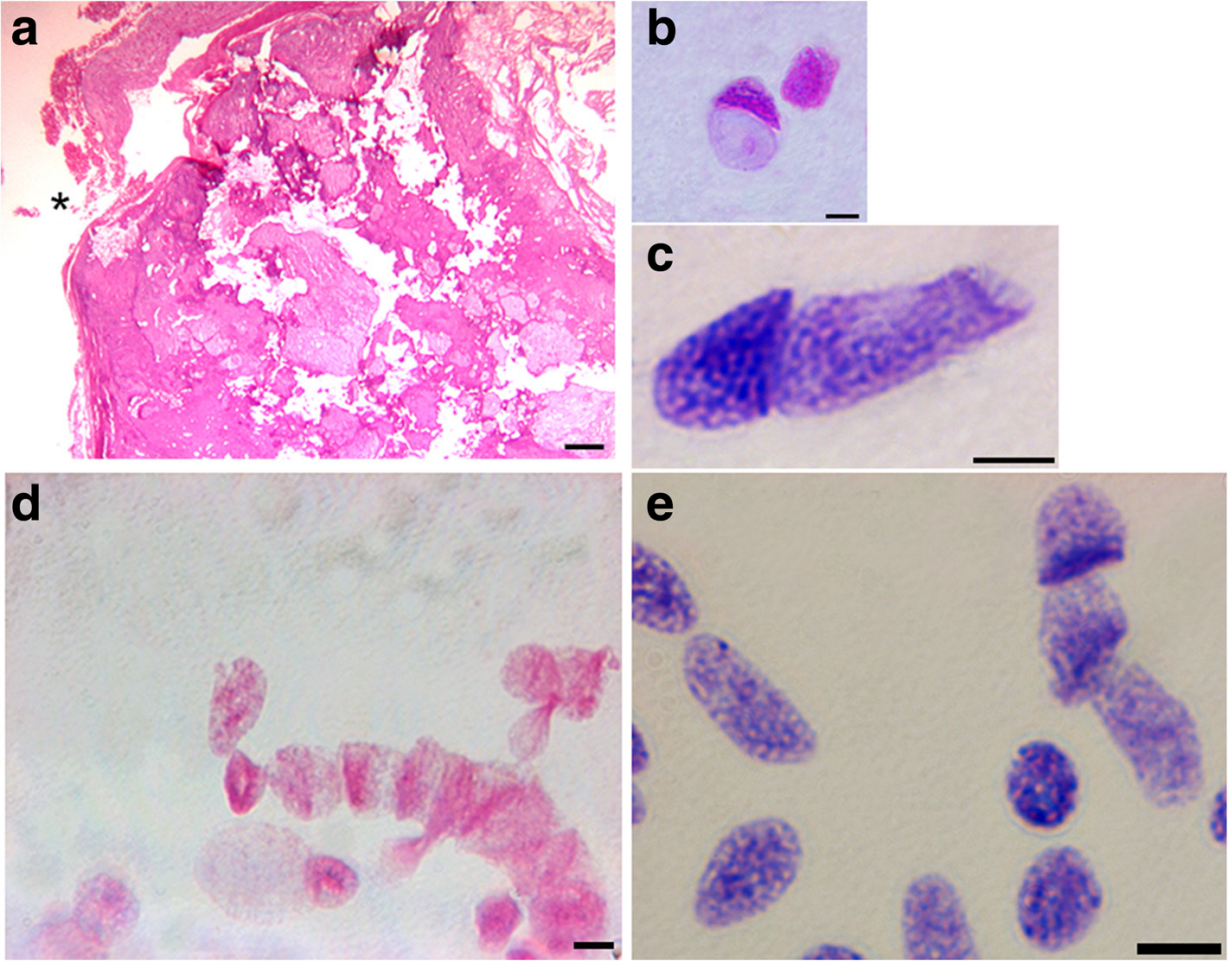
Standard 2.5 μm sections and collagenase spread of calcified tissues. **a** Haematoxylin-Eosin staining of adult calcified carotid arteries, scale bar 400 μm. **b**-**c** Feulgen staining of spread tissue of adult calcified carotid arteries showing examples of metakaryotic nuclei. **d**-**e** Feulgen staining of spread tissues showing nuclei in syncytium (fetal femoral tissue), scale bar 5 μm

Figure 5a shows a standard histological staining of the carotid plaques section (#1); of note the rupture of the endothelium (marked with an asterisk) and the presence of extensive calcification. However, the spread of the same sample (#1) allowed the identifications of a disclosed and complex cellular content. Besides the majority of the cells that have a spindle shaped nucleus (Fig. 3b), cells with a particular balloon shaped nucleus were identified in the calcified carotid plaque (in 2 cases, Fig. 5b and c). Also, we noticed that Ki-67 antigen stained some of the metakaryotic nuclei in the mononuclear forms (data not shown). The same cells were also arranged in a tubular form that reminds syncytial structure (Fig. 5d). In syncytium, Ki-67 highlighted only the cells located in the extremities (Fig. 3e and f) and nuclei inside syncytia were negative to Ki-67. This type of cells, named metakaryotic cells [12], with a typical hollow bell-shaped nucleus, was found in our study in all fetal tissues both in the syncytial form (Fig. 5d) and in the mononuclear form (Fig. 5e) while in the adult arteries, these cells were spotted in 6 cases.

## Discussion

The spreading technique showed a good reproducibility and accuracy in the evaluation of cell protein expression and single-cell DNA content in calcified carotid arteries. The IHC markers were selected in order to check the integrity of the different cellular compartments: nucleus (Ki-67) and cytoplasm (Desmin, Vimentin, Osteocalcin). To verify the reliability of the techniques we used fetal tissues sample as positive control since this tissue was already validated [12]. As standard for the Feulgen DNA quantification we chose cRBC, since they are convenient, easy to isolate and they have a constant known DNA content [18, 19]. Our results confirmed the reliability of the standard as cRBC genome size was proportional to the area of the cells and CV% was low (23 %). The digestion times with collagenase requested by the spreading technique varied considerably according to the different tissues (see Table 1). Nevertheless, the adult and fetal DNA content distribution curves were all Gaussian with the same trend, showing that collagenase did not influence the DNA content, regardless the digestion times. The Carnoy-fixed spread tissue also allows to extract preserved RNA, without any contaminations by proteins or phenols, and the sigmoid-shaped curves obtained at RT-PCR showed a typical PCR gene amplification.

The technique could also give new information regarding the study of calcification and osteogenesis. Usually DNA quantity is evaluated in the whole tissue; unfortunately one disadvantage is the loss of information on the single cell profile. Moreover standard decalcification procedures degrade the DNA content in calcified arteries. First this protocol avoids the use of formalin to fix the arteries and also avoids the use of strong acid to decalcify the arteries. This allows extracting directly from the slides of spread tissue the RNA and DNA and evaluates the gene expression in calcified tissues from a very small quantity of sample. Moreover the protocol preserved the morphology of the nucleus and allowed the visualization of the nuclear organization. As already reported, some cases of atherosclerotic carotid tissues contain bone lacunae-like mature structure in development with lamellar bone. In these cases, the protocol is of great help to open the “core” of the bone structure and visualize the cellular content. It is very difficult with the standard histological techniques to obtain a monolayer of cells. Surprisingly our data revealed and confirmed that the calcified part of arteries contains a heterogeneous population of cells composed by smooth muscle cells, osteoclasts-like cells and osteoblasts. Moreover the IHC revealed that some of the cells, composing the core of the calcification, were actively dividing. The observation is in line with other studies suggesting that arterial calcification is an active process [5].

Feulgen on calcified arteries spread allows spotting the peculiarity of the single cell. For example, we observed that two cases of fetal bone (#5 and #6) showed the lowest DNA absolute quantities. By reviewing the literature only a few groups spotted this paradox in DNA content. Solari et al. reported that osteoclasts undergo amitotic division by a budding mechanism in vitro [20]. Then Sundaram in 2004 demonstrated the existence of a set of cells dividing through neosis [21] and in 2014, Thilly et al. showed that the cells in syncytia divide by an amitotic mechanism with a dsRNA/DNA intermediate [22]. As Feulgen only stains DNA and not RNA [11, 13] we can hypothesize that the nuclei in the syncytia of fetal bone tissue undergo dsRNA/DNA intermediate during replication, thus explaining the lower mean quantity of DNA (1 pg to 3 pg) and the negativity to Ki-67.

Of fluctuations of DNA, defined as an increased range of DNA content, were observed both in the cell populations and within the single cells. The lowest CV variation was found in the healthy soft sample (#3) characterized by a homogenous population of SMC. Conversely the major CV in adult tissue was counted in the calcified tissue. This is likely to reflect a heterogeneous cell population in the fetal and adult pathologic tissues, compared to the cytological homogeneity of the smooth muscle cells in healthy arteries.

In conclusion, thanks to the preservation of the nuclear morphology the technique allowed to disclose osteoclasts-like cells and others cells type with a bell-shaped nucleus, recognised as metakaryotic cells [23]. These particular cells are very difficult to identify due to the loss of nuclear morphology when using strong acid for decalcification. Mainly studied in the progression of tumours [12], we can hypothesis that these cells are also a key player in atherosclerosis [24].

## Conclusion

The spread gently preserves the original morphology of each single cell in the calcified vascular tissues. The presented protocol will be used to study the calcification or osteogenic process occurring during atherosclerosis and to evaluate the single cell DNA content on a large series of calcified plaques. The opportunity to extract and amplify intact nucleic acid from the cells of heavily calcified arteries opens the possibility to study calcification on a larger scale. This is not doable when arteries are treated with decalcification reagents.

## Abbreviations

CEACAM 5, carcinoembryonic antigen-related cell adhesion molecule 5; cRBC, chicken red blood cells; CT, threshold cycle; CV, content variation; DCV, DNA content variation; EDTA, ethylenediaminetetraacetic acid; F-DNA, Feulgen DNA; GUSB, beta-glucuronidase; HCl, chloride acid; IOD, the integrated optical density; OCN, osteoclacin; OD, optical density; OSX, osterix; PKD, proteinase K buffer; RT, room temperature

## Additional files

Additional file 1: Raw data of the DNA quantity (ng) and area (um^2^) for each single cell evaluated in the study. (XLSX 77 kb) Additional file 2: Feulgen image analysis densitometry protocol with automatic measurement program wizard. (PDF 55 kb)
