# Supplementary material for: The study of calcified atherosclerotic arteries: an alternative to evaluate the composition of a problematic tissue reveals new insight including metakaryotic cells
Source: BMC Clin Pathol. 2016 Jul 29;16:12. doi: 10.1186/s12907-016-0036-6 (PMC4966787; doi:10.1186/s12907-016-0036-6)
Supplement: Additional file 2: — Feulgen image analysis densitometry protocol with automatic measurement program wizard. (PDF 55 kb) [file 12907_2016_36_MOESM2_ESM.pdf]

Additional file 1: Table S1. **Feulgen image analysis densitometry protocol with automatic measurement program wizard**.

	STEP	Operation	Comments	Window
**1**	Image enhancement	Alignment - Exposure – White balance -	Optical enhancement including brightness, contrast, and gamma correction	LIVE
**2**	Multidimensional image acquisition	Exposure (the same as step 1 - Set all channel into the dynamic range – start – measure – save as xxx in folders – start taking picture	Camera set up and saving parameters. When you capture picture always use the window 6D and never the Live window for DNA quantification. It is important to organize al images in separate folders	6D acquisition
**3**	Set Automatic Measurement Program Wizard	Open an image – Measure – Automatic multidimensional measurement – new program.	Generate the program that allows you to analyses images.	Wizard
		Select: Sigma filter NO – Shading Correction NO – Edge Enhancement NO.		Brightness, contrast, and gamma correction.
		Select the object of interest with the pick up tools and then open “advanced” options: refine selection with the histogram and then set up as follow:	Segmentation is the most important part: you separate the nuclei you want to measure from the background	Segmentation

		Deletion of artifacts: remove - Fill holes YES - Automatic object separation YES - Mode Erosion/Dilation YES - Watersheds NO		Interactive processing of the measurement mask
		Set measurement properties: Region feature (for a nucleus) - Field feature (for all area) - Draw features	Set measurement properties	Definition of a Measurement Condition
		Measures Features: Densitometric SUM, Densitometric AREA Densitometric SD	Chose Measures Features and check selected objects for artefacts / overlapping.	Measurement
**4**	Run Automatic Measurement Program	Measure menu – Run Automatic Multidimesional program -Execute	Execute a program you have generated for any number of images saved.	Run Automatic Measurement Program
